# Comparing neural activity of older adults with and without Parkinson’s disease using mobile electroencephalography in different environments

**DOI:** 10.1002/alz.089255

**Published:** 2025-01-09

**Authors:** Samantha Marshall, Gianna Jeyarajan, Jennifer Hanna Al‐Shaikh, Raphael Gabiazon, Tia Seleem, Lindsay S Nagamatsu

**Affiliations:** ^1^ Western University, London, ON Canada

## Abstract

**Background:**

Parkinson’s disease is a progressive neurodegenerative disorder that is becoming more prevalent as the population ages. Mobile neuroimaging has made it possible to observe cortical activity in this population outside of standard laboratory environments. Yet, few studies have explored the portability of these devices in a true real‐world environment without a specific task imposed on participants (e.g., dual task, motor demands).

**Method:**

Mobile electroencephalography (EEG) was utilized to examine and compare cortical activity during sitting in different environments across older adults with and without Parkinson’s disease. We used the Muse S Generation 2 Brain‐Sensing Headband to record brain function. The first condition involved participants sitting in a standard laboratory environment while their brain activity was recorded. In the second condition, participants sat in an indoor space that included a living green wall, natural light, and other people while their brain activity was recorded. Fast Fournier Transform (FFT) analysis was performed on the raw EEG data to obtain corresponding waves for each electrode to examine power (uV^2) at each frequency (Hz).

**Result:**

Preliminary findings demonstrate significant differences in mean cortical activations across older adults with and without Parkinson’s disease. Differences in cortical activations were also observed when comparing the laboratory and real‐world environment.

**Conclusion:**

These findings suggest that elicited brain activity may differ across older adults with and without Parkinson’s disease and across different environments. These findings expand current knowledge on Parkinson’s disease, brain function, and cognition using real‐world methods and technology, which may inform interventions to increase quality of life among this population. As our findings suggest that environmental factors may modulate cortical activity, we also highlight the potential and importance for real‐world methods to supplement standard research practices to increase the ecological validity of studies conducted across the scientific community.

